# Shoot traits and their relevance in terminal drought tolerance of chickpea (*Cicer arietinum* L.)

**DOI:** 10.1016/j.fcr.2016.07.016

**Published:** 2016-10

**Authors:** Purushothaman Ramamoorthy, Krishnamurthy Lakshmanan, Hari Deo Upadhyaya, Vincent Vadez, Rajeev Kumar Varshney

**Affiliations:** a International Crops Research Institute for the Semi-Arid Tropics (ICRISAT), Patancheru, India; b Jawaharlal Nehru Technological University Hyderabad (JNTUH), Hyderabad, India; c Department of Agronomy, Kansas State University, Manhattan, KS 66506, United States; d UWA Institute of Agriculture, University of Western Australia, Crawley, WA 6009, Australia; e School of Plant Biology and Institute of Agriculture, The University of Western Australia, WA, Australia

**Keywords:** Canopy temperature depression, Crop growth rate, Field phenotyping, Partitioning coefficient, Specific leaf area

## Abstract

Chickpea is the second most important legume crop largely grown under semi-arid tropics where terminal drought is one of the major constraints for its productivity. A trait-based selection had been considered more beneficial in drought tolerance breeding to overcome the environmental influence on drought yields. Large number of traits had been suggested in literature, with less indication on their importance and priority, for use in such breeding programs resulting in poor utilization of critical traits in drought tolerance breeding. To identify the most critical traits that contribute to grain yield under drought, 12 chickpea genotypes, with well-defined drought response, were field evaluated by sampling at regular intervals during the cropping period. Large range of variation was observed for shoot biomass productivity, specific leaf area (SLA) and leaf area index (LAI) at different days after sowings (DAS), canopy temperature depression (CTD) at mid-reproductive stages, growth duration and both morphological and analytical yield components. Grain yield under drought was closely associated with the rate of partitioning (p), crop growth rate (C), CTD, phenology, LAI at mid-pod fill stage, pod number m^−2^ at maturity, shoot biomass at reproductive growth stages and SLA at physiological maturity. The shoot trait(s) were prioritized based on their significance and contribution to drought tolerance. The trait(s) that conferred tolerance varied across genotypes. The order of traits/plant functions identified as important and critical for the drought tolerance were p, C, CTD, growth duration and other related traits. Relatively less important traits were LAI, SLA at the mid reproductive stage and pod number per unit area at maturity. The traits Dr, seeds pod^−1^ and 100-seed weight were found to be least important. Breeding for the best combination of p and C with the right phenology was proposed to be the best selection strategy to enhance terminal drought tolerance in chickpea.

## 1 Introduction

Chickpea is the second most important pulse crop world–wide, with a production of 14.2 million tons from an area of 14.8 million ha and a productivity of 0.96 t ha^−1^ (FAOSTAT, 2014). About 90% of this crop is grown rain-fed under receding soil moisture conditions in the post-rainy season after the main rainy season by resource-poor farmers (Kumar and Abbo, 2001). The crop growing environment is characterized with varying intensities and distribution of crop season rainfall from almost nil (Johansen et al., 1994) to >400 mm (Berger et al., 2004). Terminal drought of varied intensities is, therefore, a primary constraint to chickpea productivity. Drought stress (DS) alone causes substantial annual yield losses up to 50% in chickpea (Sabaghpour et al., 2006), which equaled to a loss of US $ 900 million, and the productivity remained constant for the past six decades (Ryan, 1997, Ahmad et al., 2005, Bantilan et al., 2014). By 2050, global demand for chickpea is projected to be 18.3 Mt compared to the production of 14.2 Mt in 2014, and the low-income food-deficit countries are expected to suffer the widest supply–demand gap (Nedumaran and Bantilan, 2013). This situation emphasizes the urgent need to develop drought tolerant cultivars for an increased productivity.

Breeding for drought tolerance, using the available chickpea germplasm resources, had provided various genotypes that are early in flowering and escape terminal drought effects thereby ensuring average grain yields and yield stability. Though the drought escape strategy is successfully exploited by the farmers by improving the yield stability considerably (Kumar and Abbo, 2001), this may fail to utilize the extended growing period when available (Ludlow and Muchow, 1990, Johansen et al., 1997). In order to raise the average grain yield productivity and to narrow down the supply-demand gap, development of drought tolerant/avoiding cultivars is mandatory. Moreover, such drought tolerant genotypes have been identified in the past by screening accessions of chickpea germplasm, on the basis of yield under DS, that were known to come from drought-prone areas (Saxena, 1987, Saxena, 2003, Saxena et al., 1993, Krishnamurthy et al., 2010). However, to achieve a stable and consistent drought tolerance across environments, constitutive traits or traits that are closely associated with the grain yield under DS need to be considered as a selection criterion rather than grain yield itself, as grain yields are prone to large G × E interaction (Ludlow and Muchow, 1990). Also, a trait-based breeding increases the probability of crosses resulting in additive gene action (Reynolds and Trethowan, 2007, Wasson et al., 2012). However, the list of such contributing traits proposed in literature remains very many and unmanageable (Araus, 1996, Richards, 1996, Mitra, 2001, Reynolds, 2002, Ribaut, 2006, Serraj et al., 2009, Hopkins et al., 2009, Jain et al., 2010) requiring rationalization and ranking of these traits on importance (Richards, 1996, Huang et al., 2006, Rauf and Sadaqat, 2008).

For better success in drought tolerance breeding, the traits of choice need to be causal rather than the effect (Kashiwagi et al., 2006a) and an integrator of the responses to events across the whole life cycle e.g., transpiration efficiency (TE) and partitioning coefficient (Krishnamurthy et al., 2013a, Krishnamurthy et al., 2013b). Crop models help in dissecting the grain yield into its components that can be considered more generic and organizationally close to the yield. One such model splits the grain yield as a function of three component traits, viz, crop growth rate, reproductive duration and partitioning coefficient (Duncan et al., 1978, Williams and Saxena, 1991) that are easy to measure in large populations. Also the components of this model are shoot-based and are amenable for selection through other surrogate traits.

Crop growth rate (C) is an integrated expression of both transpiration and transpiration efficiency. Recognition of its importance for drought tolerance, breeding for C had been extensively practiced in wheat and groundnut (Calderini et al., 1997, Wright et al., 1993). Large-scale field measurements of transpiration and transpiration efficiency are cumbersome. Therefore, surrogate traits for transpiration such as leaf area index (LAI) (Fageria et al., 2010) and canopy temperature depression (CTD) (Fuchs and Tanner, 1966, Jackson et al., 1981, Fuchs, 1990, Jones, 1992, Jones et al., 2002, Jones et al., 2009, Rebetzke et al., 2013) and for transpiration efficiency, carbon isotope discrimination, specific leaf area index and SPAD chlorophyll meter readings were sought to breed for in various legume crops (Comstock and Ehleringer, 1993, Sheshshayee et al., 2006, Thompson et al., 2007, Nageswara Rao et al., 2001, Bindu-Madhava et al., 2003, Kashiwagi et al., 2006a, Arunyanark et al., 2008). High heritability and a weak response to environmental variation of harvest index (HI) (Hay, 1995) had made HI suitable as a major trait for improving yield stability under DS. However, HI alone had not been considered as a yield determining trait for selection as high yields under DS were the product of interaction of both C and HI. An independent selection for HI alone was considered to pose the danger of selecting entries with a poor plant biomass potential (poor C) (Wallace et al., 1993). Therefore, success in selecting for high yield under DS requires a simultaneous selection for both C and HI. HI is a product of two components; i.e. the reproductive duration (Dr) and the rate of partitioning (p) to grains (Duncan et al., 1978, Williams and Saxena, 1991, Gallagher et al., 1976, Scully and Wallace, 1990, Krishnamurthy et al., 1999). Terminal DS in chickpea, as in many other crops, is known to reduce the growth duration, especially the reproductive phase (Krishnamurthy et al., 2013a). Chickpea growing environments experience a ceiling to the reproductive growth duration due to progressively increasing terminal DS and heat stress at the final stages of reproductive growth, requiring an increased p, thereby providing the plants to escape the later stress stages with less adverse effects on the yield formation (Krishnamurthy et al., 2013a). Several plant functions such as increased radiation use efficiency (RUE), non-lodging crop stands, increased sink size (twin pods in each node or smaller leaf size), more terminal branches, synchrony in flowering and greater flower production per unit area can be envisaged as contributing to increased p.

In addition there are several other shoot traits such as photosynthetic efficiency, chlorophyll, content, chlorophyll refraction, ABA content, proline accumulation, stomatal conductance etc. were also been proposed for use in selecting for drought tolerant genotypes. Measuring all the model components and the closely-related major traits under field condition was expected to reveal the level of contribution to grain yield and drought tolerance.

It is not only the shoot traits but also the root traits, their ability and pattern of soil water extraction that are known to contribute to drought tolerance (Cutforth et al., 2013, Bandyopadhyay, 2014, Lynch et al., 2014). The capacity of various root traits to confer yield advantages under DS and their ranking in importance of conferring drought tolerance from this set of studies have been listed such as RLD → RDp → RSR (Purushothaman et al., 2016a). Also the soil water uptake, development of drought stress across the whole growth period and the association of soil water uptake with the rooting density across soil horizon in relation to the genotypes and their drought tolerance have been already described (Purushothaman et al., 2016b). Therefore, in order to complete the series the objectives of this paper were (1) to assess the variation in shoot traits of chickpea with variable drought responses across crop growth stages and drought treatments (2) to assess the shoot traits association with the grain yield under drought and (3) to rank the traits in the order of their importance in conferring drought tolerance to chickpea enabling a targeted drought tolerance breeding.

## 2 Materials and methods

### 2.1 Plant material and crop management

Twelve chickpea genotypes viz., ICC 4958, ICC 8261, ICC 867, ICC 3325, ICC 14778, ICC 14799, ICC 1882, ICC 283, ICC 3776, ICC 7184, Annigeri, and ICCV 10 with close phenology but good contrasts for root development, drought response and canopy temperature (CT) were chosen for this study were field-evaluated on a Vertisol (fine montmorillonitic isohyperthermic typic pallustert) during the post-rainy season, in 2009–2010 and 2010–2011, at ICRISAT, Patancheru (17°30′N; 78°16′E; altitude 549 m) in peninsular India. The water holding capacity of this field in lower limit: upper limit was 0.26:0.40 cm cm^−1^ for the 0–15 cm soil layer, and 0.30:0.47 cm cm^−1^ for the 105–120 cm soil layer. The available soil water up to 120 cm depth was 165 mm, and the bulk density was 1.35 g cm^−3^ for the 0–15 cm soil layer and 1.42 g cm^−3^ for the 105–120 cm soil layer (El-Swaify et al., 1985). The field used was solarized using a polythene mulch during the preceding summer primarily to fully protect the crop from wilt causing fungi *Fusarium oxysporum* f. sp, among other benefits and damages (Sharma et al., 1988).

The fields were prepared in to broad bed and furrows with 1.2 m wide beds flanked by 0.3 m furrows. Surface application and incorporation of 18 kg N ha^−1^ and 20 kg P ha^−1^ as di-ammonium phosphate were carried out. The experiment was conducted in a randomized complete block design (RCBD) with three replications. Seeds were treated with 0.5% Benlate^®^ (E.I. DuPont India Ltd., Gurgaon, India) + Thiram^®^ (Sudhama Chemicals Pvt. Ltd. Gujarat, India) mixture for both 2009–10 and 2010–11 seasons. The seeds were hand-sown manually at a depth of 2–3 cm maintaining a row to row distance of 30 cm and a plant to plant distance of 10 cm with in rows with a row length of 4 m on 31 October, 2009 and 20 November, 2010. About 82 seeds were used for each 4 m row and at 10 days after sowing (DAS) the plants were thinned maintaining a plant-to-plant spacing of 10 cm. A 20 mm irrigation through sprinklers was applied immediately after sowing to ensure uniform seedling emergence. Subsequently, plants were grown under two soil water treatments; rainfed (to impose terminal DS) and optimal irrigation (irrigated once in 15–20 days on the basis of previous experience). The plots were kept weed free by hand weeding and intensive protection were taken against pod borer (*Helicoverpa armigera*).

### 2.2 Canopy temperature measurement

Deviation of temperature of plant canopies in comparison to ambient temperature, known as CTD has been recognized as indicators of overall plant water status (Ehrler, 1972, Blum et al., 1982, Jackson et al., 1981, Idso, 1982) and had been largely used to evaluate plant responses to drought stress (Blum et al., 1989, Royo et al., 2002, Rashid et al., 1999) and cultivar comparison for water use (Pinter et al., 1990, Hatfield et al., 1987). Higher CTD (positive value) at reproductive duration is found to be one of the selection criterion for drought tolerance. Therefore, the thermal images of plant canopies were captured about once in three days from 63 DAS onwards, when all the genotypes reached the early to mid-podding stage under DS, by an infrared camera, IR FLEXCAM (Infrared Solutions, Inc, USA) with a sensitivity of 0.09 °C and an accuracy of ±2% between 1400 and 1445 h (when maximum VPD is known to occur) from a height of 1.0 m above the canopy. The target area of the image obtained was about 30 × 20 cm at the center of each plot, and the images were captured from north to avoid shading of the target area (Kashiwagi et al., 2008). The software SmartView 2.1.0.10 (Fluke Thermography Everett, WA, USA) was used for eliminating the ground area reflection and for analyzing the images and the estimation of CT and canopy proportions following the previous report by Zaman-Allah et al. (2011). Based on the mean CT recorded in any one frame the CTD (=air temperature (T_a_) − canopy temperature (T_c_)) was calculated.

### 2.3 Soil moisture measurement

The TRIME-soil moisture probe was used to measure the available soil moisture content in the field. TRIME access tubes, with a depth of 150 cm and an inner diameter of 4.2 cm, were installed in each plot and the measurements were taken in both the OI and DS to measure the soil water depletion rate during the cropping season (Supplementary Fig. S1; Purushothaman et al., 2016b).

### 2.4 Periodical crop growth measurement

Chickpea plants were harvested, from an area of 0.75 m^2^,in each plot to comprehend the shoot biomass variation in each genotype. Such samplings were done at 28 (mid-vegetative stage), 51 (early reproductive stage), 84 (close to maturity under DS) and 96 DAS (close maturity of the optimally irrigated crop) in 2009–2010. These samplings in 2010–11 were at 24 (mid-vegetative stage), 37 (late vegetative stage), 48 (early reproductive stage), 58 (mid-reproductive stage), 70 (late reproductive stage) and 80 DAS (close to maturity). The plant components leaf, stem and reproductive parts were separated and dried in a hot-air oven at 70 °C till there were no weight change and the leaf dry weight (LDW), stem dry weight (StDW) and the reproductive parts dry weight were recorded. Besides the dry weights of the components, specific leaf area, leaf area index were also measured.

### 2.5 Specific leaf area (SLA)

The separated compound leaves were placed between two plastic transparent sheets and scanned and the scanned image was used to measure leaf area (LA) by using an image analysis system (WinRhizo, Regent Instruments INC., Quebec, Canada). The leaf samples were then oven-dried to measure leaf dry weight. The SLA was calculated as (=Leaf area (cm^2^)/Leaf dry weight (g)).

### 2.6 Leaf area index

Total LA per square meter ground area was estimated using the leaf harvested from the sampled ground area (0.75 m^2^). WinRhizo software was used to estimate the LA of the sample harvested. The LAI was calculated as (=Leaf area (m^2^)/Ground area (m^2^)).

### 2.7 Crop phenology

By regular observation, the date when 50% or more of the plants in a plot flowered was recorded as days to 50% flowering time of the plot and when 80% of the pods in a plot were dried was recorded as days to maturity for each plot.

### 2.8 Final harvest

At maturity, plant aerial parts (shoot − fallen pinnules) were harvested from an area of 3.6 m × 8 rows in each plot in both the year. Total shoot dry weights of the harvested sample were recorded after oven drying till constant weight at 45 °C in draught air driers and the total shoot dry weights were recorded. This shoot dry weight was adjusted for an estimated 20% loss of dry matter as pinnule fall (Saxena, 1984, Williams and Saxena, 1991). Grain weights were recorded after threshing. HI (%) was calculated as 100 × (grain yield/total shoot biomass at maturity). Plants from 0.75 m^2^ area were used for the estimation of pod number and seed number m^−2^, seed number pod^−1^ and their weights. 100-seed weight was estimated from these seed weight and numbers.

### 2.9 Crop growth rate, reproductive duration and partitioning coefficient

The time taken for the crop pre-flowering and post-flowering periods was converted to thermal time using temperatures recorded at the meteorological observatory of ICRISAT Asia center. Base temperature (t_b_) was taken to be 0 °C (Williams and Saxena, 1991, Singh and Virmani, 1996) and the equation used for calculating thermal time (°Cd) was:

$$\text{Cd}=\overset{n}{\underset{t=0}{\sum }}\left(\ldots -{\text{t}}_{\text{b}}\right)\frac{{\text{t}}_{\text{max}}+{\text{t}}_{\text{min}}}{2}$$

The crop growth rate (C) in kg ha^−1^ °Cd and p of each genotype were estimated using the equations:

C = (V + Y)/(Dv + Dr)

and p = (Y/Dr)/C

where: V  = Vegetative shoot mass kg ha^−1^ (adjusted for pinnule fall)

Y  = Grain weight kg ha^−1^

Dr  = Duration of growth after the start of 50% flowering °Cd

Dv  = Duration of growth before the start of 50% flowering °Cd

### 2.10 Statistical analysis

The data recorded for all the phenotypic traits at different crop growth stages in 2009–2010 and 2010–2011 were subjected to one way ANOVA. Significance of means was estimated through F value for each trait. The means derived from the ANOVA were used for correlations, regressions using GenStat software (12th edition) and path coefficient analysis using MINITAB^®^ Release 14.1 software. Variance components due to genotypes (σ^2^_g_) and error (σ^2^_e_) and their standard errors were determined. Here, the treatment (drought) was treated as a fixed effect and the genotype (G) × treatment (T) interaction as random. The variance due to (G) (σ^2^_g_) and G × T interaction (σ^2^_gT_) and their standard error were determined. Broad sense heritability (h^2^) was estimated as h^2^ = σ^2^_g_/(σ^2^_g_ + (σ^2^_e_/r)) where r was the number of replications.

## 3 Results

### 3.1 Weather and drought patterns

In both the years, the rain received prior to the cropping season was >900 mm, well distributed and more than enough to ensure complete charging of the soil profile. Cessation of seasonal rainfall occurred at 3rd October in 2009–10 and 15thNovemeber in 2010–11. In-season rains summed up to 44 mm during 9–19 DAS in 2009–10 and 12.6 mm during 19–22 DAS in 2010–11 which delayed the onset of drought slightly but the terminal drought stress did built up (data not shown). There was another rain (39 mm) at 75 DAS during 2009-10, but at this stage under drought stress the early or medium maturing accessions crossed the stage of responsiveness. Overall, the minimum temperatures were higher, particularly during the critical third and fourth week of December (flowering and early-podding period), and maximum temperatures were lower during 2009–10 (Supplementary Fig. S2). Relatively cooler minimum temperatures and maximum temperatures at vegetative period were observed in 2010–11. The cumulative evaporation and VPD was higher in 2009–10 compared to 2010–11 (Supplementary Fig. S2).

Largely, the pattern and the rate of soil moisture depletion remained the same in both the years but the soil moisture depletion was very rapid in 2010–11 season in the initial two weeks as a result of high soil evaporation and a marginally high VPD (Supplementary Fig. S2). However, the rain that followed at 18–22 DAS minimized the soil moisture depletion. Also this year the soil moisture at harvest was slightly high. There was a large rain at 75 DAS in 2009–10 which raised the surface soil moisture to some extent, benefitted the late genotypes under DS and adversely affected all the genotypes under optimally irrigated treatment but this reverted to the usual dry condition within two weeks.

### 3.2 The extent of variation in shoot traits

Genotypes varied in shoot biomass, SLA and LAI measured at different stages in both drought treatments and years (Table 1). The trial mean of shoot biomass, SLA and LAI of genotypes across drought treatments were close at the first sampling (28 DAS) in 2009–10 and (24 DAS) in 2010–11 as there was no additional irrigation provided for the optimal irrigation (OI) treatment. The OI treatment received first irrigation at 38 DAS in 2009–10 and 30 DAS in 2010–11. The genotype × drought treatment interaction effect was significant for shoot biomass at the reproductive stage in 2009–10 (Appendix A).

DS decreased the shoot biomass production mainly at the reproductive stages of crop growth than the vegetative stage. Under DS, the shoot biomass produced at 51 DAS in 2009–10 was two-folds high compared to the shoot biomass produced at 48 DAS in 2010–11 due to the rain received at 18 and 19 DAS, enhancing the shoot biomass production (Table 1). Moreover, this effect was also seen mainly in LAI than in SLA indicating that irrigation seems to increase the leaf number extensively than its area. There were wide range of variations among genotypes for shoot biomass under DS and it widened further with the increasing crop age. In addition, the range of variation among genotypes was high in 2009–10 at 84 DAS (131.3 g m^−2^) compared to 2010–11 at 80 DAS (95.6 g m^−2^). The variation among the genotypes for shoot biomass measured at all the different DAS was significantly different at a p ≤ 0.001 in both the years under DS. The same pattern was also seen under OI in both the years except at 58 and 70 DAS in 2010–11. In both years under DS, every genotype produced moderate or high shoot biomass than the trial mean at some stage of crop growth except for ICC 7184 in 2010–11 as its shoot biomass was lower than the mean across all growth stages. Terminal drought progression was more normal in 2010–11 than in 2009–10 as there were some rainy episodes that intervened with the drought setting in 2009–10. Consequently, genotypic discrimination for shoot biomass and LAI were found to be relatively high in 2010–11. At the vegetative stage in both the years under DS, genotypes ICC 4958, ICC 8261 and Annigeri have produced high shoot biomass than the mean, and ICC 1882, ICC 14778, ICC 14779, ICC 3325 and ICC 7184 produced lower shoot biomass than the mean (Table 4, Table 5). At reproductive stage, ICC 4958, Annigeri, ICC 8261, ICCV 10, ICC 14778 and ICC 14799 have produced high shoot biomass than the mean, and ICC 3776 and ICC 7184 have produced lower shoot biomass than the mean. Across all the crop growth stages, genotypes ICC 4958, ICC 8261 and Annigeri have produced high shoot biomass and, ICC 7184 has produced low shoot biomass compared to the mean. In summary, the shoot biomass production and LAI were not high for the highly drought tolerant (ICC 14778), drought tolerant (ICC 14799 and ICC 3325) and drought sensitive genotypes at vegetative stage. But, such a lead developed at the reproductive stage for the drought tolerant genotypes while the drought sensitive genotypes continued to have low shoot biomass across stages.

The heritability of shoot biomass was high and ranged from 0.421 to 0.824 in 2009–10 and 0.680–0.863 in 2010–11 under DS, and from 0.474 to 0.823 in 2009–10 and 0.279–0.849 in 2010–11 under OI (Table 1). SLA ranged from 0.038 to 0.116 in 2009–10 and 0.203–0.646 in 2010–11 under DS, and from 0.040 to 0.197 in 2009–10 and 0.164–0.637 in 2010–11 under OI. LAI ranged from 0.060 to 0.503 in 2009–10 and 0.660–0.853 in 2010–11 under DS, and from 0.153 to 0.602 in 2009–10 and from 0.055 to 0.606 in 2010–11 under OI.

### 3.3 The extent of variation in CTD

Large range of variation among the accessions for CTD was found at all times of observations. Also the genotypic variation among the accessions was different at p ≤ 0.001 in all the sampling times, across drought treatments and years. The genotype × drought treatment interaction was significant for CTD measured at 81 DAS in 2009–10 (Appendix A). Though the range and the heritability of the CTD under DS at 81 DAS in 2009–10 and 82 DAS in 2010–11 was relatively high, this observation needs to be considered with caution as some of the accessions like ICC 4958 and Annigeri had already matured and had a warmer canopy whereas others were still relatively cooler. As a consequence this final sample recorded the highest values and range in canopy temperature.

Under DS, (i) high range of genetic variation, (ii) best differentiation (widest range) in CTD, (iii) prevalence of challenging stress opportunity (as indicated by the highly negative overall mean CTD at the sampling time), and high heritability (Table 2) had occurred at both 66 and 70 DAS in 2009–10 and at 70DAS in 2010–11. Most of these performances reduced at 76DAS in 2009–10 and 72 DAS in 2010-11, respectively. Under OI, the range of genetic variation, differentiation in CTD, heritability (Table 2) indicated no clear change pattern as seen under DS. However, the high range of genetic variation, best differentiation in CTD and high heritability had occurred at 70 DAS in 2009–10 and 63 DAS in 2010–11. Since the maturity was delayed by 15–20 days, OI environment seems to provide extended periods of time for CTD sampling when the periods proximal (before and after) to irrigation were avoided.

Irrigations can reduce the canopy temperatures to a great extent and these differences can narrow down with the time after irrigation. Irrigation had brought down the canopy temperature, by 10.5 °C two days after irrigation at a stage of 81 DAS in 2009–10 and by 8.1 °C six days after irrigation at 82 DAS in 2010–11 compared to the DS crop. In contrast, it had brought down the canopy temperature only by 3.7 °C 12 days after irrigation at 76 DAS in 2009–10 and 17 days after irrigation by 3.5 °C at 72 DAS in 2010–11, thus indicating that the level of canopy cooling can vary to a great extent depending upon the time of canopy temperature sampling in relation to the irrigation time.

### 3.4 The extent of variation in crop phenology, shoot biomass, grain yield and its components

The overall means for each drought treatment across years showed that DS reduced the days to 50% flowering and days to maturity (Table 3). Overall, the DS hastened flowering by 5 days in 2009–10 and by 7 days in 2010–11 and the less hastening in 2009–10 was due to the early stage rainfall and the delayed stress buildup. Whereas DS hastened maturity by 21 days in 2009–10 and by 13 days in 2010–11. Genotypes varied significantly in days to 50% flowering and days to maturity both in 2009–10 and 2010–11. ICC 4958 and Annigeri were the earliest, ICC 283 and ICC 1882 were little longer than the early ones in crop phenology. The remaining genotypes were medium in duration. The genotype × drought treatment interaction was found to be significant for crop phenology in both the years (Appendix A). The heritability values were high for the days to 50% flowering and for days to maturity under DS whereas it turned out to be less and moderate when irrigated. This was mostly due to a rain that was received immediately after the last irrigation causing variations due to excessive vegetative growth and lodging in some genotypes.

Under DS, both the shoot biomass and the grain yield produced at maturity were slightly higher during 2009–10. DS reduced the grain yield by 4 and 45% and the shoot biomass by 46 and 47% at maturity during 2009–10 and 2010–11 seasons, respectively (Table 3). The meager reduction in grain yield in 2009–10 is more due to a poor irrigation response caused by a rainfall immediately following the last irrigation. Highly significant variations were found for the shoot biomass as well as grain yield among the genotypes, except for shoot biomass in 2009-10, and these variations were about 1.5-fold for the shoot biomass at maturity and 2-fold for grain yield among the accessions tested under DS. Under OI these variations were about 1.2–1.3 fold for the shoot biomass (Table 3). The genotype × drought treatment interaction was found to be significant for grain yield in the year 2010–11 (Appendix A).

Under DS, the genotypes that produced greater shoot biomass were the early and strong rooting kabuli ICC 8261, the drought tolerant ICC 14778 and the drought sensitive ICC 3776. Additionally, only in 2010-11, two other drought tolerant genotypes ICC 867 and ICC 3325 and the well adapted genotype ICCV 10 produced greater shoot biomass (Table 6, Table 7). Early and weak rooted ICC 283 and the best adapted Annigeri have produced the least shoot biomass across the years. The genotypes that produced consistently greater grain yield under DS were the two drought-tolerant genotypes ICC 867 and ICC 14778 and the best adapted genotype ICCV 10. Early large rooting ICC 4958, drought tolerant ICC 3325 and another best adapted genotype Annigeri yielded higher only in 2010–11. And the genotypes that produced consistently lesser grain yield under DS were the two drought-sensitive genotypes ICC 3776 and ICC 7184 along with the kabuli ICC 8261.

Heritability indices were high for the grain yield and moderate for shoot biomass under both drought treatments and year (Table 3). In general, the HI was relatively poor under OI. In 2009–10 a mean HI of 47.9 under DS was reduced to 26.6 under OI. Similarly in 2010–11, it was 45.5 under DS compared to 43.8 under OI, indicating that DS enhanced the HI compared to OI in both the years and the enhancement was much higher in 2009–10 primarily due to over watering under OI. The genotypic distribution for HI followed similar pattern as that of the grain yield under both drought treatments and years (Table 6, Table 7). The variation among the genotypes for HI was significant at <0.001 level and the heritability were also high across drought treatments and years (Table 3).

The pod number m^−2^ was low in 2009–10. Under DS pod number m^−2^ was 562.2 in 2009–10 compared to 807.2 in 2010–11. Under OI it was 675.1 in 2009–10 compared to 1420 in 2010–11. There was a huge range of variation among genotypes for pod number m^−2^. The year wise difference and the genotypic variation of seed number m^−2^ were closely similar to the pod number m^−2^ trend. The seed pod^−1^ followed a directly opposite trend compared to the traits pod number m^−2^ and seed number m^−2^ as DS slightly improved the seed pod^−1^ compared to the OI in both the years. There were minimal differences between the drought treatments for 100-seed weight in both the years. The variations among the genotypes for pod number m^−2^, seed number m^−2^, seed pod^−1^ and 100-seed weight were significant at p ≤ 0.001 level and the heritability were also high across drought treatments and years. Moreover, the genotype × drought treatment interaction was found to be significant for pod number m^−2^ and 100-seed weight in the year 2010–11 (Appendix A).

DS reduced Dr and C but increased the p (Table 3). In 2009-10, the Dr, C and p were 938.2, 2.29 and 0.852 under DS compared to 1332, 3.42 and 0.413 under OI, respectively. In 2010–11 these were 954.4, 2.40 and 0.745 under DS compared to 1157, 3.79 and 0.694 under OI, respectively. The genotype × drought treatment interaction was found to be significant for Dr and p in the year 2010–11 (Appendix A).The heritability values ranged from moderate to high for Dr and C, and high for p across drought treatments and years.

### 3.5 Shoot traits contribution to grain yield

**Fig. 1:**
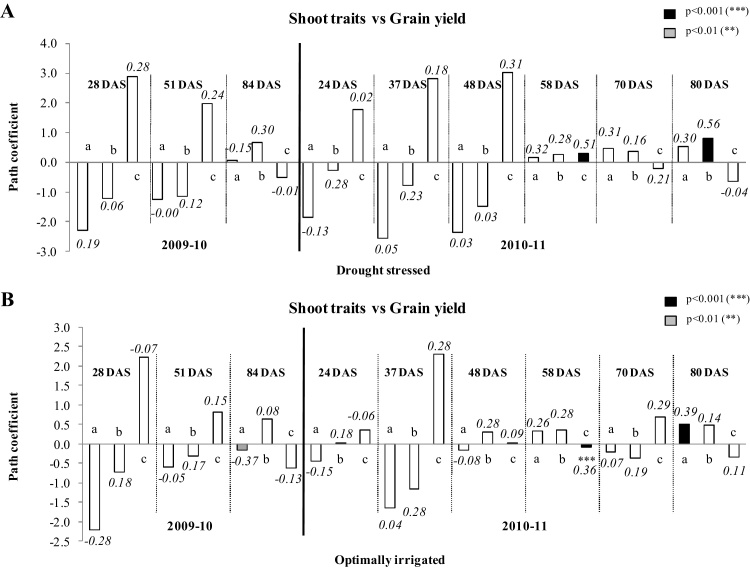
Direct effect of shoot biomass (a), specific leaf area (b) and leaf area index (c) measured at different days after sowing (DAS) on grain yield at maturity of 12 diverse genotypes of chickpea both under (A) drought stressed and (B) optimally irrigated conditions in a Vertisol during 2009–10 and 2010–11 post-rainy seasons. The different filling colors of bars denote different levels of significance of correlations between various shoot traits and grain yield. The correlation coefficient value of each trait with the grain yield associations were mentioned on the top of each bar.

In 2009-10, the direct contribution of shoot attributes measured at peak vegetative (28 DAS), early pod filling (51 DAS) and at near maturity stages (84 DAS) on grain yield was not consistent and changed from positive to negative depending on the crop growth stage (Fig. 1A and B). In 2009–10 under DS at 28 DAS, the correlation coefficients of all the shoot traits with the final grain yield were positive and nonsignificant but under OI these coefficients were nonsignificant and negative except for the SLA association.

In 2009–10 under DS, though the direct effect path coefficients of SBM and SLA were substantially negative, the total contribution had turned positive through the major direct positive contribution by LAI (Fig. 1A). Under OI, SLA had exhibited a trend of positive correlation coefficient with grain yield though its direct effect was negative (Fig. 1B). This change was caused by LAI through its positive contribution making the total contribution of SLA to grain yield positive. At 51 DAS, the pattern of contribution and direct effects of shoot traits on grain yield were similar as seen at 28 DAS sampling with a few exceptions under both OI and DS. Also, the contribution of LAI and SLA to the grain yield had remained to be high under DS than under OI. At 84 DAS, when most genotypes were near maturity under DS, the contribution of LAI to grain yield become negative under both drought treatments as these genotypes that retained more leaves were relatively longer in duration and poorer in grain yield. SLA had contributed the highest in both direct contribution and indirectly through LAI to the grain yield. Under DS, though the direct contribution of SBM to grain yield was positive, the correlation coefficient had turned negative by the influence of greater negative indirect effect of LAI (data not shown).

In 2010–11, all the shoot traits measured at various growth stages (24, 37, 48, 58, 70 and 80 DAS) showed largely nonsignificant positive correlation coefficients with the grain yield except for SBM at 24 DAS and LAI at 80 DAS, as these were negative in correlation coefficient under DS (Fig. 3A and B). Under OI, this correlation was negative with SBM and LAI at 24 DAS. Generally these correlation coefficients became positive and larger with the advance in growth stage. SBM after 58 DAS showed larger correlation coefficients particularly under DS though these were marginally short of significance. LAI at 58 DAS was closely and positively correlated with grain yield under both drought treatments. SLA at 80 DAS under DS was closely correlated with the grain yield. Under DS, LAI alone had a positive direct contribution to grain yield among the other shoot traits till 58 DAS and SBM and SLA had a clear negative direct contribution. But the contribution pattern of all these three components reversed from 58 DAS. Under OI, the direct positive contribution of SBM and SLA was highest at 80 DAS though such a trend has got set in since 58 DAS onwards.

### 3.6 Contribution of CTD to grain yield

**Fig. 2:**
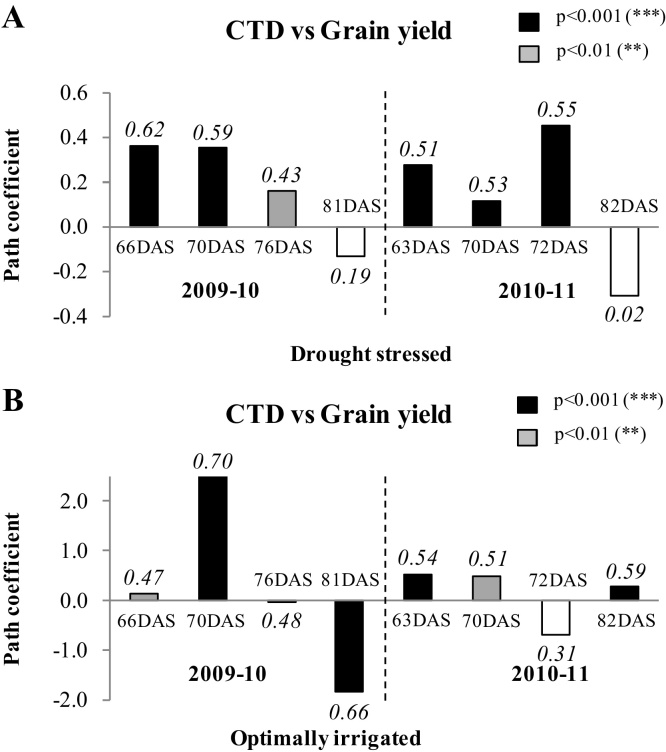
Direct effect of canopy temperature depression (CTD) measured at different days after sowing (DAS) on grain yield at maturity of 12 diverse genotypes of chickpea both under (A) drought stressed and (B) optimally irrigated conditions in a Vertisol during 2009–10 and 2010–11 post-rainy season. The different filling colors of bars denote different levels of significance of correlations between CTD and grain yield. The correlation coefficient value of CTD with the grain yield associations were mentioned on the top of each bar.

In 2009-10, the correlation coefficients of the CTD were positive at all the samplings under both drought treatments and highly significant except at 81 DAS (Fig. 2A and B). Under DS, the positive direct contribution of CTD was the highest at 70 DAS, followed by at 66 DAS. Under OI, the positive direct contribution of CTD was highest at 70 DAS with a significance level of p ≤ 0.001. In addition, the CTD at 76 and 81 DAS were also significantly correlated with grain yield at <0.01 and <0.001 levels, respectively. Though the direct contribution of CTD to grain yield was highly negative at 81 DAS, the large positive indirect contribution of 70 DAS had resulted in a positive association with grain yield at this stage.

In 2010–11, the correlation coefficients of the CTD were positive at all the samplings under both drought treatments except for the 82 DAS sample under DS (Fig. 2A and B). Under DS, the positive direct contribution CTD was highest at 72 DAS, followed by 63 DAS. Under OI, the positive direct contribution of CTD was highest at 63 DAS, followed by 70 and 82 DAS with the significance level ranging from p ≤ 0.01 to p ≤ 0.001.

In both the years, under DS, the CTD of initial three samples have had highly significant correlations with the grain yield. And this significance had extended even up to the last sample under OI.

### 3.7 Contribution of crop phenology to grain yield

**Fig. 3:**
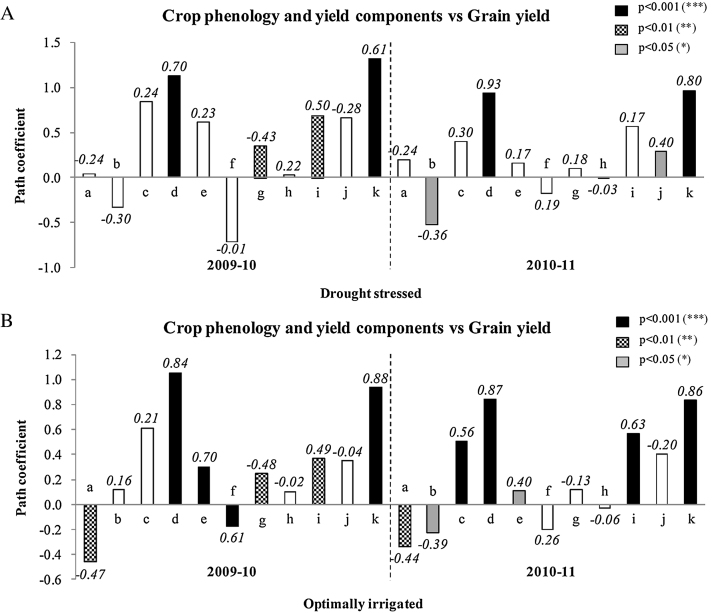
Direct effect path coefficients of days to 50% flowering (a), days to maturity (b), shoot biomass at maturity (c), harvest index (d), pod number m^−2^ (e), seed number m^−2^ (f), seeds pod^−1^ (g), 100-seed weight (h), crop growth rate (i), reproductive duration (j) and partitioning coefficient (k) on grain yield at maturity of 12 diverse genotypes of chickpea both under (A) drought stressed and (B) optimal irrigation in a Vertisol during 2009–10 and 2010–11 post-rainy seasons. The different filling colors of bars denote different levels of significance of correlations between various shoot traits and grain yield. The correlation coefficient value of each trait with the grain yield associations were mentioned on the top of each bar.

Crop phenology (days to 50% flowering and the maturity) was negatively correlated with grain yield across drought treatments and years except for days to maturity under OI in 2009–10 (Fig. 3A and B). Under DS, the days to 50% flowering had positive direct contribution to grain yield and the days to maturity had a high negative contribution to it, explaining the negative correlation coefficients in both the years (Fig. 3A). Under OI, the days to 50% flowering had a negative direct contribution to grain yield and the correlations were significant at p ≤ 0.01 in both the years (Fig. 3B). The days to maturity showed a positive direct contribution in 2009–10, and a high negative direct contribution to grain yield in 2010–11. The correlation of days to maturity with grain yield was significant at p ≤ 0.05. The phenological reactions under OI in 2009–10 was different due to the rain following the last irrigation.

### 3.8 Contribution of shoot biomass, HI and morphological components to grain yield

In terms of association with the grain yield or by contribution to grain yield, the yield components shoot biomass at maturity, HI and pod number m^−2^ were important. The other three yield components, seed number m^−2^, seeds pod^−1^ and 100-seed weight has had minimum contribution or role in grain yield determination (Fig. 3A and B). There were trends of positive association of shoot biomass at maturity with grain yield irrespective of the drought treatments but it was significantly correlated only under OI in 2010–11. HI was very closely associated with grain yield in both irrigation regimes and years and also the contributions were positive and large at all environments. Pod number m^−2^ was also positively correlated whereas it was significant under both drought levels only in 2010–11. Seed number m^−2^ was also positively correlated whereas it was only significant under DS in 2010–11. Seeds pod^−1^ was negatively correlated whereas it was only significant in 2009–10 under both drought regimes. 100-seed weight was not generally correlated but for the indication of positive association under DS in 2009–10.

Under DS in both the years, shoot biomass at maturity had a large positive direct contribution to grain yield but this did not result in significant correlation (Fig. 3A) mainly due to an influence of large negative indirect effect by HI (data not shown). Higher shoot biomass production, in many of the later maturing genotypes, did not reflect in grain yield by the poor partitioning. The path coefficient of HI showed a high direct positive and a highly significant contribution to grain yield at p ≤ 0.001 (Fig. 3A). This was possible due to the indirect contribution of pod numbers per unit area (data not shown). The occurrence of large negative direct effect of seed number m^−2^ results in a nonsignificant correlation with grain yield. Seeds pod^−1^ had a positive direct effect on grain yield which could not affect the correlation mostly due to negative indirect contribution of seed number m^−2^. 100-seed weight had a small positive contribution that was largely suppressed by the negative indirect contribution by pod number m^‐2^ and seeds pod^−1^ (data not shown). Also under OI, closely similar pattern of association of all the shoot traits to the final grain yield can be seen (Fig. 3B). But the major difference was the absence of major negative indirect contribution of HI to shoot biomass and therefore the shoot biomass association was significant with final grain yield. But the direct contribution of shoot biomass itself was low compared to the DS.

In summary, in both the years and drought treatments, the HI had a consistent direct positive contribution as well as a highly significant correlation with grain yield. In addition, the shoot biomass, pod number m^−2^ also often had a consistent positive direct contribution leading to a significant correlation with grain yield with some exception.

### 3.9 Contribution of analytical components to grain yield

In both the years and drought treatments, the analytical component p had the closest association with grain yield explaining the highest levels of yield variation. Also this trait had provided the best positive direct contribution to the grain yield (Fig. 3A and B). The other two components provided a negative indirect contribution to grain yield through p (data not shown).

In both the years and drought treatments, the analytical component C had a close association with grain yield except under DS in 2010–11. Also C had provided a positive large direct contribution to the grain yield across drought environments and years (Fig. 3A and B). The component p tend to provide a major negative indirect contribution to grain yield under DS while Dr provided a major negative indirect contribution to grain yield under OI (data not shown).

In both the years and drought treatments, the analytical component Dr had a loosely negative, mostly nonsignificant, association with grain yield except under DS in 2010–11. But Dr had provided a positive large direct contribution to the grain yield across drought environments and years (Fig. 3A and B). The component p tended to provide a major negative indirect contribution negating the positive contribution of Dr to grain yield (data not shown).

## 4 Discussion

### 4.1 Shoot traits contribution to drought tolerance

Under DS, the extent of shoot biomass produced at vegetative growth stages negatively influence grain yield but the shoot biomass produced at reproductive stages tend to have positive influence. But when irrigated no such clear influence on grain yield was noticeable. The composition of the genotypes in this study and the large positive effects of LAI explain this effect. The early large rooting genotypes, ICC 4958 and ICC 8261, produced the best shoot biomass at the early stages but their grain yield was the least in ICC 8261 and not the best in ICC 4958 due to its early phenology fixing a ceiling on the potential yield and limits the crop's ability to exploit extended growing periods (Serraj et al., 2004). Also the vegetative stage LAI had a massive positive influence through shoot biomass on grain yield but the final association turned to be neutral. However, at reproductive stages the direct effects of both the shoot biomass and the SLA turned largely positive and their associations with the grain yield was significant. However, all these discussions indicate that vegetative stage shoot biomass is not the single trait to concentrate in drought tolerance breeding (Arradeau, 1989, Bidinger and Witcombe, 1989, Olaoye, 1999). Greater shoot biomass at maturity had been recognized to lead to greater grain yields and greater drought tolerance in chickpea (Rosales-Serna et al., 2004, Fenta et al., 2012, Kashiwagi et al., 2015) and greater biomass partitioning to grains had been found to produce the best drought tolerance (Duncan et al., 1978, Scully and Wallace, 1990, Rao, 2001, Beebe et al., 2008, Davies et al., 2000, Ainsworth et al., 2011, Jogloy et al., 2011, Krishnamurthy et al., 2013a).

SLA had responded to DS with a reduction as an adaptive measure and with no genotypic deviation. But there were large genetic variation for SLA. The highly drought tolerant genotype ICC 867 had the highest SLA at all the growth stages and drought sensitive genotypes had the least with very few exceptions. But a clear genetic aligning of SLA with drought reaction had not been noticeable. SLA offered substantial direct negative effects on grain yield at the vegetative stages that changed to direct positive effects at the reproductive stages. But the correlations improved with the advances in growth stage to become significant at close to maturity only in the intense DS year. SLA is well known to be a drought tolerance indicator in many crops (Nigam et al., 2005, Arunyanark et al., 2008, Jongrungklang et al., 2008, Pimratch et al., 2008) but a smaller SLA is considered to be advantageous in groundnut facing more of an intermittent DS (Wright and Hammer, 1994, Nageswara Rao et al., 2001, Painawadee et al., 2009) and other crops (Brown and Byrd, 1996, Pita and Pardos, 2001, Ciordia et al., 2012). A lower SLA becomes advantageous for a less water loss and a more C exchange ensuring plant survival. But under terminal drought stress as seen here in chickpeas the strategy seemed to be different. Drought tolerant leaf expansion seemingly lead to a greater leaf area and a greater drought tolerance (De Costa, 1998).

DS had reduced LAI by almost half during the reproductive stages of crop growth and there were no drought × genotypes interaction. Also there had been a large genetic variation for SLA. Unlike SLA, no clear association of LAI with drought reaction was noticeable at any growth stages. LAI offered large direct positive effects on grain yield at the vegetative stages that turned into a positive association once at 58 DAS both under DS as well as under OI. LAI is an adaptive trait. Plants lose their leaves rapidly to get adjusted to the soil water environments well. However, it is clear that amongst the three shoot traits used for testing their contribution to grain yield LAI can be rated as the most important.

### 4.2 Contribution of CTD to drought tolerance

CTD is a crop response to drying soils and environment. This functional aspect cannot be rated as a trait but can be considered as an integrated response of both the roots ability for soil water acquisition and the stomatal conductance (Jackson et al., 1981, Jones et al., 2002, Jones et al., 2009, Lopes and Reynolds, 2010, Rebetzke et al., 2013). Its application and use had been recent but it had been well accepted as a reliable selection tool to assess the overall plant water status, continuance of stomatal conductance and canopy transpiration. Under DS best differentiation (widest range) in CTD, large number of genotypes exhibiting highly negative CTDs (warmer canopies) as an indication of suffering the consequences of water deficit and a close association of CTD with drought yields had been listed desirable at the time of sampling for the best estimate of drought yields or drought tolerance (Zaman-Allah et al., 2011, Belko et al., 2012, Rebetzke et al., 2013). Its usefulness as a selection tool had been well demonstrated also in chickpea but appearance of such an association had been temporal (Purushothaman et al., 2015). The CTD measurements made at different times had been brought together for a separate path analysis to propose the best time of measurements. In this study, the best association of CTD with grain yield has been seen to occur at both 66 and 70 DAS in 2009–10 and at 63, 70 and 72 DAS in 2010–11. Such an association started to disappear from 76 DAS onwards in 2009–10 and 82 DAS in 2010–11. In wheat, CTD has been found to be associated with not only the grain yield but also with shoot biomass and HI at the reproductive stage (Rebetzke et al., 2013). The best adapted genotypes Annigeri and ICCV 10 maintained a CTD close to the mean at all the stages of samplings except for an insignificant increase at 82 DAS in 2010–11. Previous findings had supported the inference that active root functioning at this stage had been responsible for the cooler canopy and a greater drought avoidance (Purushothaman et al., 2015). Such suggestions of active water extraction, through prolific and deep root systems, playing a major role in keeping the canopy cooler for a longer time also had been made in other crop species (Kashiwagi et al., 2008, Lopes and Reynolds, 2010, Rebetzke et al., 2013). The CTD of ICC 4958 was clearly lower than the mean from 70 DAS in 2009–10 and 72 DAS in 2010–11 (data not shown). This early large rooting genotype was the shortest in duration and escaping the major part of the terminal DS (Saxena, 1987, Gaur et al., 2008, Kumar and Abbo, 2001). When the measurements were done this genotype was already in an advanced stage of growth approaching maturity with the root and shoot partly senesced that led to the lower CTD or warmer canopy. But this was an artifact of ‘delayed observation’ as far as ICC 4958 is concerned. However, ICC 4958 displayed other characteristics for rating it as a successful drought tolerant genotype.

The contribution of CTD to grain yield under OI, did follow a similar pattern of genetic variation but the OI mean remained high (or the canopy was substantially cooler) compared to the DS indicating the constituent nature of CTD influencing traits. Based on the significant association with grain yield, CTD measured at 70 DAS in 2009–10 and 63 DAS in 2010–11 found to be the most suitable time for estimating grain yield. In wheat, while screening for heat tolerance, 10 days after anthesis was found to be the critical time for the best discrimination of genotypes through their CTD differences (Gowda et al., 2011). Since the maturity was delayed by 15–20 days by OI, enhanced soil moisture seems to provide an extended period of time for measuring CTD when the periods that are proximal (before and after) to irrigation were avoided.

### 4.3 Contribution of crop phenology, shoot biomass and harvest index to drought tolerance

DS reduced both the number of days to 50% flowering and days to maturity compared to OI (Krishnamurthy et al., 2013a) as observed in many other crops such as in soybean (Desclaux and Roumet, 1996), wheat and barley (McMaster and Wilhelm, 2003). The length of both these phenological stages had a negative contribution to grain yield, and the contribution was found to be significant for days to maturity indicating lesser the reproductive duration greater the grain yield. In soybean, severity of water deficit at anthesis had been found to reduce grain yield significantly through increased pod abortion (Liu et al., 2003).The genotypes used in this study contain both early and medium duration ones. Genotypes that are early in duration had been considered to fit well with the available season and the quantity of available soil water (Saxena, 1987, Gaur et al., 2008, Kumar and Abbo, 2001). However, the growing duration of highly tolerant genotypes were slightly longer than the early ones, and were capable of yielding higher by using the available extended growing period (Johansen et al., 1997, Bolanos and Edmeades, 1996, Krishnamurthy et al., 2010).

An increased shoot biomass production at maturity is considered to be one of the key requirements for drought tolerance (Krishnamurthy et al., 1999, Krishnamurthy et al., 2013a, Krishnamurthy et al., 2013b, Serraj and Sinclair, 2002, Richards et al., 2002). The direct effect of path coefficient and the correlation between shoot biomass and grain yield in this study was positive but didn’t attain a significant level mainly due to the indirect influence of HI and pod number m^2^ under DS. Moreover, consistency of HI’s association with grain yield indicates its importance across environments (Viola, 2012, Fischer and Edmeades, 2010, Krishnamurthy et al., 1999, Krishnamurthy et al., 2010, Krishnamurthy et al., 2013a, Krishnamurthy et al., 2013b, Rehman, 2009, Ribaut et al., 2009). Increased shoot biomass with equally greater HI had been suggested to be a drought tolerance indicator and this combination of traits was incorporated in many other studies successfully. Maintenance of higher shoot biomass production under DS was through maintenance of greater C or greater transpiration (Passioura, 1994, Kashiwagi et al., 2006b, Kashiwagi et al., 2013) and maintenance of higher HI was through efficient water use during post-flowering period by the deeper and profuse root system (Wasson et al., 2012, Krishnamurthy et al., 2013a, Kashiwagi et al., 2015).

### 4.4 Contribution of morphological yield components to drought tolerance

Among the morphological yield components, pod number m^−2^ under DS was found to have a positive association with grain yield or drought tolerance in this study. Similar positive associations had been reported not only under DS but also under other abiotic stresses like salinity (Zeng and Shannon, 2000) and heat (Krishnamurthy et al., 2011a, Viola, 2012). For example, in this study genotype ICC 14778 performed consistently greater in pod number m^−2^ than the mean across drought treatments and years. And this ability in setting increased number of pods had helped it also to be a superior genotype for the best grain yields under terminal DS and yield stability (Acosta-Gallegos and Adams, 1991, Silim and Saxena, 1993, Loss and Siddique, 1997, Rehman, 2009, Krishnamurthy et al., 2013a). On the other hand, 100-seed weight had offered a significant negative influence on pod number m^−2^ in this study balancing the pod number’s contribution. Previous researches had also suggested that increased pod number to link well with a reduced seed size under DS (Saxena and Sheldrake, 1976). It is necessary to avoid these traits’ interaction, by selecting both for increased pod number m^−2^ and higher 100-seed weight simultaneously.

### 4.5 Contribution of analytical yield components

DS had specifically reduced Dr indicating its vulnerability and can be expected to exert proportionate grain yield losses (Krishnamurthy et al., 2013a). When water is not limited for T, canopy temperatures are known to be cooler and remain close to 25 °C deviating heavily from the ambient temperatures. Cooler temperatures and shorter photoperiods are known to encourage suppression of reproductive growth (Roberts et al., 1985). Conversely, soil water deficit and enhanced temperatures had been known to hasten the reproductive processes but with a proportionately reduced final plant productivity. Selective shortening of reproductive growth phase is commonly observed not only in response to DS (Krishnamurthy et al., 2013a) but also in response to heat or salinity stress (Krishnamurthy et al., 2010, Krishnamurthy et al., 2011a, Krishnamurthy et al., 2011b). Contribution of Dr to grain yield was negative in all the environments except under DS in 2010–11. OI had increased C. However, C had a significant contribution to grain yield in both the drought treatments and years. Among the studied genotypes, large root genotypes (ICC 4958 and ICC 8261) had a high C and, the small root (ICC 1882 and ICC 283) and the drought sensitive genotypes (ICC 3776 and ICC 7184) had a lower C. The crop growth rate had been suggested to be considered as a trait for water harvesting since the total water use, viz. total T, had strong correlations with the total crop productivity (Udayakumar et al., 1998, Condon et al., 2002). Compared to the small root producing genotypes or the drought sensitive genotypes, the large rooting genotypes seemed to have the advantage of greater water extraction which reflects in an increased total T resulting in greater C under DS (Kashiwagi et al., 2015).

Analytical components Dr and p together constitute HI (Jogloy et al., 2011, Krishnamurthy et al., 1999). Therefore, any effort to maintain a higher HI under water deficit needs to aim for a much greater p to compensate for the loss of Dr so as to keep the yield gap reduced. The realization of the importance of p and the approach of selection for enhanced p or HI is not new (Adams, 1982, Duncan et al., 1978, Scully and Wallace, 1990, Jogloy et al., 2011, Krishnamurthy et al., 1999, Krishnamurthy et al., 2013a). The association of p with grain yield was close irrespective of the drought treatments or year. Also the direct contribution of p to grain yield had remained the highest leading to a high total contribution despite the large indirect contribution of C and Dr. Measurement of p is simple and any yield evaluation field trial is sufficient to record the required parameters. It is well realized that many interacting traits contribute to drought tolerance with their importance shifting with the level of stress intensity (Tardieu, 2012). The advantage of p, as a complex resultant state of various processes, is that it could be improved through many of the traits operating simultaneously. Surprisingly, this trait possesses the best h^2^ surpassing even the estimates for the phenological observations (Krishnamurthy et al., 2013a). The range of genetic variation for p was found to be high. The p of the highly drought tolerant genotype ICC 14778 and the widely-adapted genotype ICCV 10 were the highest and highly consistent explaining their superior grain yields particularly under DS. Both the drought sensitive genotypes (ICC 3776 and ICC 7184) and the kabuli genotype (ICC 8261) had the lowest p. When p was regressed with the grain yield, it explained 76–82% of the grain yield variation. This shows the constitutive nature of this trait meriting consideration in drought tolerance breeding.

Considering collectively the appearance of direct effects, closeness and consistency of the correlations with the grain yield under DS and the relatively better appearance of these responses under DS and the heritability these traits can be arranged in the following order of priority for use in drought tolerance breeding.

p (HI) → CTD → C (shoot biomass at maturity) → Phenology → LAI (mid-pod fill stage) → pod numberm^−2^ → shoot biomass (reproductive growth stages) → SLA at physiological maturity.

The traits Dr, seeds pod^−1^ and 100-seed weight did not qualify for consideration due to inconsistency in association and the lack of substantial direct effects towards grain yield or no effects and null contribution.

## 5 Conclusion

The genotypes, selected for contrasts in various drought response traits, varied widely for growth characteristics and traits but the detection of their association depended upon the growth stage of measurement and soil water status. The order of traits or the plant functions that were identified as important and critical for the drought tolerance are the rate of partitioning (closely related to HI and CTD), C (related to shoot biomass productivity at maturity) and the right phenology. Second order and relatively less important traits are a high LAI, high SLA at the mid reproductive growth stage and a large pod number per unit area at maturity. CTD or allied measuring methods in a critical stage of reproductive growth dependent on soil water can indicate drought tolerance closely. The traits Dr, seeds pod^−1^ and 100-seed weight did not show any meaningful association with drought tolerance. It is suggested that breeding for the best combination of p and C with the right phenology can result in best CTD and the grain yield under terminal drought.

## Figures and Tables

**Table:** 

	Drought treatment		Drought treatment × Genotype	
	Wald statistic	Significance level	σ^2^_g_ (SE)	Significance level
2009–10				
SBM_28DAS	10.9	0.007	0.095 (0.527)	ns
SLA_28DAS	0.590	0.459	24.4 (69.7)	ns
LAI_28DAS	2.95	0.114	0.0005 (0.0003)	ns
SBM_51DAS	26.0	<0.001	298.5 (149.9)	*
SLA_51DAS	15.4	0.002	73.0 (190.0)	ns
LAI_51DAS	18.6	0.001	0.097 (0.085)	ns
SBM_84DAS	66.1	<0.001	1000.0 (627.0)	ns
SLA_84DAS	7.41	0.020	73.0 (172.0)	ns
LAI_84DAS	153.9	<0.001	0.107 (0.171)	ns
SBM_96DAS	95.4	<0.001	7060.0 (3333.0)	*

CTD_66DAS	520.5	<0.001	0.194 (0.125)	ns
CTD_70DAS	711.1	<0.001	0.064 (0.066)	ns
CTD_76DAS	659.4	<0.001	0.035 (0.062)	ns
CTD_81DAS	977.0	<0.001	0.595 (0.291)	*

Days to 50% flowering	36.0	<0.001	3.60 (1.72)	*
Days to maturity	121.2	<0.001	20.1 (9.49)	*
Shoot biomass (kg ha^−1^)	443.4	<0.001	54325 (64830)	ns
Grain yield (kg ha^−1^)	1.94	0.191	7724 (7744)	ns
Harvest index	175.8	<0.001	12.8 (6.63)	ns
Pod number m^−2^	3.89	0.074	15676 (8408)	ns
Seed number m^−2^	3.10	0.106	10235 (5490)	ns
Seed pod^−1^	0.120	0.732	0.0057 (0.0035)	ns
100-seed weight	0.800	0.390	1.88 (0.996)	ns
Dr	185.1	<0.001	3906.0 (2174.0)	ns
C	279.6	<0.001	0.005 (0.013)	ns
p	179.8	<0.001	0.0047 (0.0028)	ns

2010–11				
SBM_24DAS	5.21	0.043	0.0006 (0.106)	ns
SLA_24DAS	30.4	<0.001	47.3 (45.6)	ns
LAI_24DAS	0.950	0.350	0.00002 (0.00006)	ns
SBM_37DAS	0.000	0.968	6.07 (3.70)	ns
SLA_37DAS	92.1	<0.001	56.4 (71.5)	ns
LAI_37DAS	7.08	0.022	0.0012 (0.0019)	ns
SBM_48DAS	7.16	0.022	13.4 (11.3)	ns
SLA_48DAS	103.0	<0.001	41.5 (93.6)	ns
LAI_48DAS	38.1	<0.001	0.0060 (0.0070)	ns
SBM_58DAS	103.3	<0.001	5.0 (18.0)	ns
SLA_58DAS	176.0	<0.001	12.2 (57.6)	ns
LAI_58DAS	150.8	<0.001	0.009 (0.013)	ns
SBM_70DAS	31.3	<0.001	263.4 (146.7)	ns
SLA_70DAS	169.6	<0.001	31.6 (84.0)	ns
LAI_70DAS	135.4	<0.001	0.019 (0.047)	ns
SBM_80DAS	162.4	<0.001	359.8 (213.9)	ns
SLA_80DAS	23.0	<0.001	147.0 (216.0)	ns
LAI_80DAS	132.3	<0.001	0.032 (0.076)	ns

CTD_63DAS	761.5	<0.001	0.036 (0.089)	ns
CTD_70 DAS	533.1	<0.001	0.011 (0.145)	ns
CTD_72 DAS	367.2	<0.001	0.097 (0.104)	ns
CTD_82 DAS	320.1	<0.001	0.932 (0.533)	ns

Days to 50% flowering	29.3	<0.001	8.82 (3.82)	*
Days to maturity	108.5	<0.001	8.33 (3.99)	*
Shoot biomass (kg ha^−1^)	397.5	<0.001	111608 (67618)	ns
Grain yield (kg ha^−1^)	199.9	<0.001	50054 (23573)	*
Harvest index	3.07	0.107	4.34 (2.37)	ns
Pod number m^−2^	44.7	<0.001	44616 (21564)	*
Seed number m^−2^	78.6	<0.001	21548 (11720)	ns
Seed pod^−1^	3.01	0.111	0.0081 (0.0045)	ns
100-seed weight	7.99	0.016	1.89 (0.96)	*
Dr	58.5	<0.001	3498.0 (1789.0)	*
C	193.9	<0.001	0.043 (0.027)	ns
p	5.16	0.044	0.0026 (0.0013)	*

**Table 1:** Trial means and analysis of variance of shoot traits of 12 diverse genotypes of chickpea at different days after sowing (DAS) both under drought stressed and optimally irrigated conditions in a Vertisol during 2009–10 and 2010–11 post-rainy season.

Year/sampling time/trait	Drought stressed					Optimally irrigated				
	Trial mean	Range of means	S.Ed	σ^2^_g_ (F pr.)	Heritability (h^2^)	Trial mean	Range of means	S.Ed	σ^2^_g_ (F pr.)	Heritability (h^2^)
2009–10, 28 DAS										
SBM	12.4	8.27–20.4	1.43	10.6 (<0.001)	0.762	13.8	9.39–21.5	1.36	14.2 (<0.001)	0.815
SLA	198.8	171.8–224.0	20.1	1.12 (0.393)	0.038	195.4	181.0–216.3	15.3	1.42 (0.233)	0.122
LAI	0.224	0.160–0.350	0.038	4.04 (0.003)	0.503	0.239	0.162–0.389	0.037	5.53 (<0.001)	0.602

2009–10, 51 DAS										
SBM	126.0	84.0–160.4	9.18	11.6 (<0.001)	0.779	164.9	123.2–220.3	11.1	15.0 (<0.001)	0.823
SLA	167.5	143.7–194.4	19.2	1.19 (0.348)	0.060	198.1	166.8–238.8	36.2	0.89 (0.567)	0.000
LAI	1.66	1.10–2.10	0.235	3.46 (0.006)	0.450	2.43	1.52–3.79	0.520	3.38 (0.007)	0.442

2009–10, 84 DAS										
SBM	265.3	199.7–331.0	32.6	3.18 (0.010)	0.421	391.9	325.3–493.6	25.6	7.17 (<0.001)	0.673
SLA	168.3	130.0–189.6	21.2	1.39 (0.243)	0.116	186.4	145.7–230.8	30.3	1.74 (0.130)	0.197
LAI	2.08	1.73–2.56	0.392	1.19 (0.348)	0.060	4.91	3.36–5.83	0.985	1.54 (0.186)	0.153

2009–10, 96 DAS										
SBM	356.6	250.3–495.3	26.1	17.3 (<0.001)	0.844	709.1	605.3–840.3	49.3	3.70 (0.004)	0.474

2010–11, 24 DAS										
SBM	7.43	5.59–12.0	0.670	19.9 (<0.001)	0.863	6.99	5.52–10.6	0.611	16.4 (<0.001)	0.837
SLA	203.5	181.0–236.0	7.50	6.47 (<0.001)	0.646	226.4	199.6–261.4	11.5	6.26 (<0.001)	0.637
LAI	0.126	0.091–0.193	0.014	12.4 (<0.001)	0.792	0.131	0.095–0.197	0.017	5.60 (<0.001)	0.606

2010–11, 37 DAS										
SBM	26.6	20.9–46.7	2.30	19.7 (<0.001)	0.862	26.7	17.1–39.8	2.10	17.9 (<0.001)	0.849
SLA	172.4	158.3–193.1	10.8	2.01 (0.079)	0.251	204.2	172.6–239.6	15.2	2.39 (0.039)	0.317
LAI	0.409	0.315–0.762	0.041	18.4 (<0.001)	0.853	0.479	0.277–0.661	0.061	5.87 (<0.001)	0.619

2010–11, 48 DAS										
SBM	52.8	43.6–69.5	4.31	7.37 (<0.001)	0.680	58.4	46.7–85.4	5.71	8.19 (<0.001)	0.706
SLA	170.3	155.6–204.2	11.4	2.69 (0.023)	0.360	227.4	185.9–268.8	26.6	1.67 (0.148)	0.182
LAI	0.763	0.572–0.988	0.075	6.89 (<0.001)	0.662	1.079	0.709–1.628	0.180	3.73 (0.004)	0.476

2010–11, 58 DAS										
SBM	94.5	67.8–118.0	7.03	10.4 (<0.001)	0.759	119.7	89.5–137.8	11.0	3.81 (0.004)	0.484
SLA	178.0	163.4–210.4	10.8	2.99 (0.014)	0.399	237.2	212.5–282.5	17.9	2.71 (0.022)	0.363
LAI	1.35	0.835–1.64	0.110	8.59 (<0.001)	0.717	2.21	1.63–2.77	0.245	3.48 (0.006)	0.452

2010–11, 70 DAS										
SBM	166.3	111.4–198.5	10.4	13.1 (<0.001)	0.801	208.6	189.9–232.5	13.4	2.16 (0.060)	0.279
SLA	186.5	157.0–212.9	16.2	1.90 (0.097)	0.203	250.6	226.1–306.4	27.0	1.59 (0.170)	0.164
LAI	1.91	1.19–2.65	0.206	10.2 (<0.001)	0.753	3.43	2.36–4.91	0.516	3.33 (0.008)	0.438

2010–11, 80 DAS										
SBM	225.5	187.0–282.6	12.7	11.5 (<0.001)	0.778	341.5	285.8–382.2	13.9	11.3 (<0.001)	0.774
SLA	171.3	142.8–197.4	12.8	3.66 (0.005)	0.470	214.2	163.8–276.2	28.2	3.17 (0.010)	0.420
LAI	1.59	0.995–2.06	0.166	6.83 (<0.001)	0.660	3.43	2.70–4.35	0.689	1.17 (0.358)	0.055

↑SBM = Shoot biomass; SLA = Specific leaf area; LAI = Leaf area index.

**Table 2:** Trial means and analysis of variance of canopy temperature depression of 12 diverse genotypes of chickpea at different days after sowing (DAS) both under drought stressed and optimally irrigated conditions in a Vertisol during 2009–10 and 2010–11 post-rainy seasons.

Year/treatment/sampling time	Canopy temperature depression				
	Trial mean	Range of means	S.Ed	σ^2^_g_ (F pr.)	Heritability (h^2^)
2009–10, Drought stressed					
66 DAS	−0.020	−2.45 to 1.03	0.533	6.21 (<0.001)	0.634
70 DAS	−0.690	−2.70 to 0.592	0.475	7.45 (<0.001)	0.683
76 DAS	−2.61	−3.82 to −1.69	0.421	4.94 (<0.001)	0.568
81 DAS	−5.77	−8.21 to −3.94	0.476	10.7 (<0.001)	0.763

2009–10, Optimally irrigated					
66 DAS	4.99	4.06 to 5.61	0.323	3.54 (0.006)	0.458
70 DAS	3.51	1.04 to 4.31	0.333	18.4 (<0.001)	0.853
76 DAS	1.08	−0.843 to 2.23	0.487	7.24 (<0.001)	0.675
81 DAS	4.76	1.84 to 5.85	0.330	23.1 (<0.001)	0.880

2009–10, Drought stressed					
63 DAS	−1.86	−4.25 to −1.08	0.465	7.29 (<0.001)	0.677
70 DAS	−2.17	−4.40 to −0.950	0.498	8.59 (<0.001)	0.717
72 DAS	−1.41	−2.62 to −0.492	0.389	5.79 (<0.001)	0.615
82 DAS	−4.78	−7.98 to −3.04	0.733	6.54 (<0.001)	0.649

2009–10, Optimally irrigated					
63 DAS	2.93	−0.117 to 4.20	0.610	7.39 (<0.001)	0.680
70 DAS	3.06	−0.557 to 5.31	0.809	7.09 (<0.001)	0.670
72 DAS	2.07	−0.143 to 3.53	0.603	5.37 (<0.001)	0.593
82 DAS	3.35	0.416 to 5.19	0.626	9.11 (<0.001)	0.730

**Table 3:** Trial means and analysis of variance of 12 diverse chickpea genotypes for phenology, shoot biomass at maturity, grain yield, harvest index, morphological and analytical yield components both under drought stressed and optimally irrigated conditions in a Vertisol during 2009–10 and 2010–11 post-rainy seasons.

Year/treatment/trait	Trial mean	Range of means	S.Ed	σ^2^_g_ (F pr.)	Heritability (h^2^)
2009–10, Drought stressed					
Days to 50% flowering	47.0	38.0–52.0	0.800	52.4 ( < 0.001)	0.945
Days to maturity	92.0	78.7–100.0	2.20	16.7 ( < 0.001)	0.839
Shoot biomass (kg ha^−1^)	3793	3395–4605	285.0	3.21 (0.010)	0.425
Grain yield (kg ha^−1^)	1795	1093–2078	102.4	13.6 ( < 0.001)	0.807
Harvest index (%)	47.9	29.1–56.4	2.29	28.6 ( < 0.001)	0.902
Pod number m^−2^	562.2	287.8–715.6	41.0	18.8 ( < 0.001)	0.856
Seed number m^−2^	641.9	282.9–910.2	49.4	26.5 ( < 0.001)	0.895
Seed pod^−1^	1.13	0.983–1.44	0.049	15.7 ( < 0.001)	0.831
100-seedweight (g)	17.5	10.4–31.9	0.930	89.9 ( < 0.001)	0.967
Dr	938.2	858.4–1050	54.1	3.16 (0.010)	0.419
C	2.29	2.07–2.63	0.153	2.39 (0.039)	0.317
p	0.852	0.501–1.04	0.072	10.2 ( < 0.001)	0.755

2009–10, Optimally irrigated					
Days to 50% flowering	51.7	49.3–54.0	1.04	4.24 (0.002)	0.519
Days to maturity	112.7	110.0–115.3	0.931	5.99 ( < 0.001)	0.624
Shoot biomass (kg ha^−1^)	7073	6171–7682	369.0	3.59 (0.005)	0.463
Grain yield (kg ha^−1^)	1871	1308–2362	149.6	9.59 ( < 0.001)	0.741
Harvest index (%)	26.6	17.4–32.2	2.12	10.1 ( < 0.001)	0.752
Pod number m^−2^	675.1	224.4–1021	102.0	12.5 ( < 0.001)	0.794
Seed number m^−2^	723.4	227.6–1027	72.5	20.0 ( < 0.001)	0.863
Seed pod^−1^	1.12	0.891–1.63	0.078	16.2 ( < 0.001)	0.835
100-seedweight (g)	17.0	8.63–29.5	0.680	183.4 ( < 0.001)	0.984
Dr	1334	1240–1433	33.6	5.77 ( < 0.001)	0.614
C	3.42	3.01–3.81	0.187	3.24 (0.009)	0.428
p	0.413	0.271–0.522	0.031	10.6 ( < 0.001)	0.762

2010–11, Drought stressed					
Days to 50% flowering	44.8	33.0–52.3	0.480	338.4 (<0.001)	0.991
Days to maturity	90.5	83.0–94.7	0.820	36.2 (<0.001)	0.922
Shoot biomass (kg ha^−1^)	3700	3198–4133	134.3	7.41 (<0.001)	0.681
Grain yield (kg ha^−1^)	1681	1078–2118	71.1	44.0 (<0.001)	0.935
Harvest index (%)	45.5	27.3–54.0	1.21	100.3 (<0.001)	0.971
Pod number m^−2^	807.2	359.3–1118	64.0	20.9 (<0.001)	0.869
Seed number m^−2^	975.1	340.0–1685	88.4	30.4 (<0.001)	0.907
Seed pod^−1^	1.18	0.893–1.51	0.077	10.4 (<0.001)	0.758
100-seedweight (g)	14.7	8.51–28.2	0.960	79.5 (<0.001)	0.963
Dr	954.4	872.5–1067	22.3	15.4 (<0.001)	0.828
C	2.40	2.11–2.59	0.090	5.39 (<0.001)	0.594
p	0.745	0.490–0.913	0.023	72.4 (<0.001)	0.960

2010–11, Optimally irrigated					
Days to 50% flowering	51.4	47.0–55.0	0.537	41.3 (<0.001)	0.931
Days to maturity	103.5	95.0–107.3	1.92	4.92 (<0.001)	0.567
Shoot biomass (kg ha^−1^)	6926	5652–7928	381.3	5.16 (<0.001)	0.581
Grain yield (kg ha^−1^)	3037	1877–4202	89.87	93.7 (<0.001)	0.969
Harvest index (%)	43.8	32.5–55.8	1.89	30.2 (<0.001)	0.907
Pod number m^−2^	1420	707.1–2162	129.6	18.2 (<0.001)	0.851
Seed number m^−2^	1574	555.1–2291	119.3	31.4 (<0.001)	0.910
Seed pod^−1^	1.11	0.8–1.43	0.057	33.8 (<0.001)	0.916
100-seedweight (g)	16.5	8.7–33.9	0.784	206.6 (<0.001)	0.986
Dr	1157	984.9–1218	49.6	3.06 (0.012)	0.407
C	3.79	3.01–4.26	0.254	4.23 (0.002)	0.518
p	0.694	0.522–0.875	0.033	25.3 (<0.001)	0.890

↑Dr = Reproductive duration (°Cd); C = Crop growth rate; p = Parititioning coefficient.

**Table 4:** Shoot growth of 12 diverse genotypes of chickpea at different days after sowing (DAS) both under drought stressed and optimally irrigated conditions in a Vertisol during 2009–10 post-rainy season.

Genotype/treatment	28DAS			51DAS			84DAS			96DAS
	SBM	SLA	LAI	SBM	SLA	LAI	SBM	SLA	LAI	SBM
Drought stressed										
ICC 4958	20.4	187.0	0.350	160.4	162.8	2.08	331.0	146.5	1.76	250.3
ICC 8261	14.5	171.8	0.216	141.7	143.7	1.71	275.8	130.0	2.19	442.3
ICC 867	13.2	224.0	0.274	132.3	194.4	2.10	298.5	188.3	2.56	313.7
ICC 3325	13.0	209.3	0.246	124.9	175.2	1.92	290.8	173.9	2.56	306.1
ICC 14778	8.3	206.7	0.160	84.0	164.9	1.10	199.7	179.8	2.07	439.0
ICC 14799	10.4	204.7	0.204	89.4	180.3	1.29	210.3	189.6	1.73	344.7
ICC 1882	8.8	194.0	0.163	114.9	165.8	1.53	214.1	160.0	1.85	341.4
ICC 283	10.6	191.4	0.189	125.0	151.3	1.52	281.2	160.2	1.92	348.7
ICC 3776	11.1	199.3	0.199	133.9	172.3	1.70	249.1	179.3	2.20	495.3
ICC 7184	10.4	217.7	0.193	127.7	180.9	1.50	296.0	159.6	2.40	421.0
Annigeri	14.8	199.7	0.268	150.5	170.7	1.94	291.2	179.5	1.97	258.9
ICCV 10	12.8	180.1	0.222	127.1	147.5	1.54	246.1	173.1	1.76	318.3

Mean	12.4	198.8	0.224	126.0	167.5	1.66	265.3	168.3	2.08	356.6
S.Ed (±)	1.43	20.1	0.038	9.18	19.2	0.235	32.6	21.2	0.392	26.1

Optimally irrigated										
ICC 4958	21.5	207.7	0.389	220.3	222.1	3.79	396.2	165.4	3.94	840.3
ICC 8261	19.4	181.0	0.303	189.0	190.3	2.86	465.3	151.8	5.81	692.7
ICC 867	12.4	212.2	0.238	123.2	196.2	1.80	381.4	230.8	5.67	759.7
ICC 3325	14.0	209.3	0.267	174.3	228.0	3.14	373.0	215.1	5.70	738.3
ICC 14778	10.4	195.8	0.185	134.0	182.6	2.00	325.3	192.8	4.71	632.7
ICC 14799	11.6	216.3	0.241	165.5	238.8	2.28	381.9	205.1	5.83	642.0
ICC 1882	9.4	195.7	0.162	129.3	210.4	2.06	334.3	220.9	5.45	605.3
ICC 283	11.7	182.4	0.191	154.8	170.8	1.91	368.3	145.7	3.36	717.0
ICC 3776	14.1	187.8	0.224	192.3	166.8	2.44	493.6	175.8	5.11	664.3
ICC 7184	12.2	184.2	0.186	137.8	176.5	1.52	372.3	182.8	4.45	769.7
Annigeri	16.4	181.6	0.264	164.9	179.7	2.20	420.7	194.3	5.20	749.7
ICCV 10	12.3	191.1	0.221	193.9	214.6	3.20	390.5	156.0	3.74	697.0

Mean	13.8	195.4	0.239	164.9	198.1	2.43	391.9	186.4	4.91	709.1
S.Ed (±)	1.36	15.3	0.037	11.1	36.2	0.520	25.6	30.3	0.985	49.3

↑SBM = Shoot biomass; SLA = Specific leaf area; LAI = Leaf area index.

**Table 5:** Shoot growth of 12 diverse genotypes of chickpea at different days after sowing (DAS) both under drought stressed and optimally irrigated conditions in a Vertisol during 2010–11 post-rainy season.

Treatment/genotype	24DAS			37DAS			48DAS			58DAS			70DAS			80DAS		
	SBM	SLA	LAI	SBM	SLA	LAI	SBM	SLA	LAI	SBM	SLA	LAI	SBM	SLA	LAI	SBM	SLA	LAI
Drought stressed																		
ICC 4958	11.00	199.6	0.186	46.7	178.3	0.762	69.5	173.0	0.988	118.0	163.4	1.43	198.5	157.0	1.59	230.5	156.1	0.99
ICC 8261	11.96	196.4	0.193	33.0	167.2	0.486	67.8	161.9	0.918	109.6	173.4	1.59	198.4	189.0	2.47	250.1	147.9	2.06
ICC 867	6.71	236.0	0.131	24.9	193.1	0.439	53.0	204.2	0.970	86.9	210.4	1.52	153.7	212.9	2.07	249.1	197.4	1.89
ICC 3325	5.62	210.4	0.101	21.8	172.7	0.340	43.6	175.4	0.665	84.7	187.9	1.33	193.6	201.7	2.65	200.9	174.5	1.58
ICC 14778	5.59	210.6	0.103	22.4	176.5	0.350	49.5	168.6	0.734	82.2	185.8	1.31	157.6	203.6	2.49	206.8	181.9	1.65
ICC 14799	6.18	210.3	0.112	20.9	187.8	0.341	50.2	184.0	0.815	78.5	186.6	1.20	167.0	187.4	2.06	190.1	192.9	1.65
ICC 1882	7.53	206.4	0.136	25.0	163.4	0.370	47.3	170.3	0.696	106.6	176.8	1.56	160.2	183.1	1.89	282.6	167.8	1.84
ICC 283	6.50	202.8	0.114	22.8	177.2	0.362	49.9	160.0	0.661	92.9	173.3	1.23	185.7	184.0	1.73	217.8	169.7	1.35
ICC 3776	6.38	181.0	0.092	23.4	158.3	0.315	49.2	155.6	0.628	86.8	167.0	1.16	139.0	186.4	1.73	187.0	163.4	1.45
ICC 7184	5.91	198.1	0.091	23.8	159.3	0.328	45.3	164.6	0.572	67.8	169.1	0.83	111.4	173.9	1.19	200.3	142.8	1.49
Annigeri	9.04	190.2	0.141	30.1	171.3	0.442	50.3	162.4	0.672	102.2	177.2	1.42	148.5	192.9	1.28	228.1	170.7	1.25
ICCV 10	6.78	200.1	0.116	24.7	164.1	0.373	58.3	163.6	0.840	117.2	165.3	1.64	182.1	165.7	1.72	262.7	190.7	1.89

Mean	7.43	203.5	0.126	26.6	172.4	0.409	52.8	170.3	0.763	94.5	178.0	1.35	166.3	186.5	1.91	225.5	171.3	1.59
S.Ed (±)	0.670	7.50	0.014	2.30	10.8	0.041	4.31	11.4	0.075	7.03	10.8	0.110	10.4	16.2	0.206	12.7	12.8	0.166

Optimally irrigated																		
ICC 4958	10.33	231.4	0.197	39.7	202.1	0.661	85.4	246.8	1.63	135.4	236.26	2.27	200.2	229.4	2.64	335.4	188.1	2.80
ICC 8261	10.61	199.6	0.173	36.4	187.2	0.589	75.1	209.4	1.31	137.8	219.4	2.39	223.2	226.1	3.44	348.4	167.7	3.43
ICC 867	5.87	253.7	0.122	21.9	213.4	0.438	58.2	233.0	1.14	105.2	253.4	2.09	189.9	270.0	3.61	307.9	276.2	3.85
ICC 3325	6.69	239.2	0.138	24.8	215.5	0.498	54.5	259.3	1.16	123.6	282.5	2.77	219.2	306.4	4.91	323.9	244.9	3.48
ICC 14778	5.69	261.4	0.128	25.3	214.5	0.481	46.7	244.0	0.91	115.9	257.2	2.35	192.9	278.2	3.91	319.4	249.4	3.98
ICC 14799	5.52	243.6	0.106	24.3	239.6	0.518	56.7	268.8	1.22	102.3	252.3	2.24	199.0	244.7	3.21	307.7	231.2	3.52
ICC 1882	7.33	214.6	0.136	30.9	209.1	0.572	55.0	227.3	1.05	136.5	235.7	2.54	232.5	258.1	4.06	381.0	235.5	3.95
ICC 283	5.97	232.4	0.118	24.1	202.7	0.422	59.1	212.1	1.03	113.6	220.4	1.83	217.8	244.8	3.31	361.4	183.6	2.82
ICC 3776	6.26	207.6	0.110	24.6	172.6	0.363	50.6	185.9	0.71	119.0	212.5	1.82	190.6	237.9	3.00	365.6	192.1	3.21
ICC 7184	5.57	209.7	0.095	17.1	193.6	0.277	48.8	201.1	0.81	89.5	214.3	1.63	210.7	226.2	2.36	285.8	206.7	3.11
Annigeri	7.56	220.7	0.134	27.8	201.6	0.508	63.0	217.3	1.10	133.1	234.0	2.27	215.2	248.6	3.47	379.3	231.1	4.35
ICCV 10	6.51	202.8	0.112	23.0	198.9	0.423	47.5	223.2	0.88	125.0	229.0	2.33	211.6	237.1	3.25	382.2	163.8	2.70

Mean	6.99	226.4	0.131	26.7	204.2	0.479	58.4	227.4	1.08	119.7	237.2	2.21	208.6	250.6	3.43	341.5	214.2	3.43
S.Ed (±)	0.610	11.52	0.017	2.10	15.2	0.061	5.71	26.6	0.180	11.0	17.9	0.245	13.4	27.0	0.516	13.8	28.2	0.690

↑SBM = Shoot biomass; SLA = Specific leaf area; LAI = Leaf area index.

**Table 6:** Phenology, grain yield, morphological and analytical yield components of 12 diverse genotypes of chickpea both under drought stressed and optimally irrigated conditions in a Vertisol during 2009–10 post-rainy season.

Treatment/genotype	Days to 50% flowering	Days to maturity	Total shoot biomass (kg ha^−1^)	Grain yield (kg ha^−1^)	Harvest index (%)	Pod number (m^−2^)	Seed number (m^−2^)	Seed number (pod^−1^)	100-seed weight (g)	Dv (°Cd)	Dr (°Cd)	C (kg ha^−1^ °Cd^−1^)	p
Drought stressed													
ICC 4958	38	79	3507	1915	54.6	384	394	1.03	27.6	879	862	2.44	0.91
ICC 8261	48	97	4605	1674	36.3	288	283	0.98	31.9	1094	1027	2.63	0.62
ICC 867	48	90	3858	2078	54.9	716	765	1.07	16.0	1094	878	2.35	1.04
ICC 3325	48	93	3480	1752	50.4	612	645	1.05	16.2	1101	932	2.07	0.91
ICC 14778	52	96	4232	2016	48.2	683	910	1.33	13.5	1180	920	2.43	0.91
ICC 14799	50	94	3844	1734	45.0	502	623	1.25	13.9	1136	919	2.26	0.83
ICC 1882	45	89	3506	1871	53.6	604	631	1.04	14.0	1035	914	2.17	0.95
ICC 283	45	87	3395	1789	52.7	700	810	1.16	13.3	1021	887	2.16	0.94
ICC 3776	49	98	4091	1628	39.9	571	622	1.09	16.7	1108	1035	2.31	0.68
ICC 7184	50	100	3756	1093	29.1	590	846	1.44	10.4	1136	1050	2.08	0.50
Annigeri	41	82	3567	1923	53.9	548	564	1.03	18.8	949	858	2.38	0.94
ICCV 10	47	93	3669	2069	56.4	549	610	1.11	18.0	1064	976	2.18	0.98

Mean	47.0	92.0	3792.5	1795.2	47.9	562.2	641.9	1.13	17.5	1066.4	938.2	2.29	0.852
S.Ed (±)	0.80	2.20	285.0	102.4	2.29	41.0	49.4	0.05	0.93	16.5	54.1	0.15	0.072

Optimally irrigated													
ICC 4958	49	111	7116	1894	26.7	487	432	0.89	29.5	1122	1337	3.50	0.41
ICC 8261	53	115	7529	1308	17.4	224	228	1.01	28.7	1207	1361	3.55	0.27
ICC 867	51	111	7348	2158	29.2	749	793	1.07	16.9	1158	1311	3.60	0.45
ICC 3325	51	113	6846	2086	30.8	1013	855	0.89	15.6	1151	1363	3.30	0.47
ICC 14778	54	112	6404	2035	32.2	815	1027	1.27	12.6	1219	1267	3.12	0.52
ICC 14799	53	113	7378	1842	25.0	563	725	1.29	12.7	1207	1298	3.56	0.40
ICC 1882	51	114	6578	1949	29.8	1021	915	0.90	15.5	1151	1390	3.13	0.45
ICC 283	51	113	6935	1982	28.9	819	909	1.12	14.0	1165	1340	3.36	0.45
ICC 3776	53	110	7653	1529	20.0	536	707	1.31	11.6	1194	1239	3.81	0.33
ICC 7184	53	112	6171	1309	21.2	319	520	1.63	8.6	1201	1277	3.01	0.34
Annigeri	50	114	7233	1993	27.6	678	709	1.05	20.8	1144	1388	3.46	0.42
ICCV 10	50	115	7682	2362	30.7	877	861	0.99	17.1	1144	1432	3.61	0.46

Mean	51.7	112.7	7072.7	1870.5	26.6	675.1	723.4	1.12	17.0	1171.7	1333.6	3.42	0.413
S.Ed (±)	1.04	0.93	369.0	149.6	2.12	102.0	72.5	0.08	0.68	22.2	33.6	0.19	0.031

↑Dv = Vegetative duration; Dr = Reproductive duration; C = Crop growth rate; p = Partitioning coefficient.

**Table 7:** Phenology, grain yield, morphological and analytical yield components of 12 diverse genotypes of chickpea both under drought stressed and optimally irrigated conditions in a Vertisol during 2010–11 post-rainy season.

Genotypes/treatment	Days to 50% flowering	Days to maturity	Total shoot biomass (kg ha^−1^)	Grain yield (kg ha^−1^)	Harvest index (%)	Pod number (m^−2^)	Seed number (m^−2^)	Seed number (pod^−1^)	100-seed weight (g)	Dv (°Cd)	Dr (°Cd)	C (kg ha^−1^ °Cd^−1^)	p
Drought stressed													
ICC 4958	33	83	3680	1905	51.8	593	526	0.89	25.3	709	1008	2.59	0.73
ICC 8261	52	95	4133	1131	27.3	359	340	0.96	28.2	1074	920	2.51	0.49
ICC 867	47	90	3871	1878	48.6	692	856	1.24	13.4	989	896	2.49	0.85
ICC 3325	49	92	3907	1894	48.5	868	973	1.12	12.2	1011	917	2.45	0.84
ICC 14778	52	93	3822	1911	50.0	1118	1685	1.51	10.8	1074	888	2.36	0.91
ICC 14799	51	92	3639	1694	46.5	926	1171	1.26	12.0	1047	873	2.30	0.85
ICC 1882	43	93	3636	1797	49.4	915	1013	1.11	12.5	914	1030	2.26	0.77
ICC 283	41	86	3198	1535	48.0	884	1002	1.13	11.6	857	926	2.17	0.76
ICC 3776	47	94	3698	1355	36.5	682	916	1.34	10.0	979	999	2.26	0.60
ICC 7184	44	91	3339	1078	32.3	1051	1254	1.19	8.5	928	982	2.11	0.52
Annigeri	35	87	3554	1873	52.7	764	812	1.06	16.9	747	1067	2.37	0.74
ICCV 10	44	90	3921	2118	54.0	833	1154	1.39	15.2	921	947	2.54	0.88

Mean	44.8	90.5	3699.8	1680.7	45.5	807.2	975.1	1.18	14.7	937.6	954.4	2.40	0.75
S.Ed (±)	0.48	0.82	134.3	71.1	1.21	64.0	88.4	0.08	0.96	8.9	22.3	0.09	0.02

Optimally irrigated													
ICC 4958	47	103	6582	3141	47.8	1042	867	0.83	31.0	984	1218	3.62	0.71
ICC 8261	55	107	6740	2183	32.5	707	555	0.78	33.9	1123	1191	3.53	0.52
ICC 867	51	103	7215	3205	44.5	1770	1749	0.99	14.4	1052	1158	3.95	0.70
ICC 3325	53	104	7277	3174	43.6	1473	1605	1.09	14.9	1091	1137	3.95	0.71
ICC 14778	54	103	6345	3134	49.4	1700	2291	1.36	10.6	1097	1113	3.47	0.81
ICC 14799	54	105	7928	3161	39.9	1523	1891	1.24	12.1	1097	1156	4.26	0.64
ICC 1882	49	95	6918	3194	46.3	2162	1718	0.80	14.8	1017	985	4.22	0.79
ICC 283	49	104	6436	3094	48.4	1729	1992	1.15	13.2	1017	1202	3.51	0.74
ICC 3776	53	106	7205	2485	34.5	1203	1683	1.39	10.2	1080	1191	3.84	0.54
ICC 7184	53	106	5652	1876	33.2	1116	1594	1.43	8.7	1080	1191	3.01	0.52
Annigeri	50	103	7280	3597	49.6	1342	1318	0.98	18.8	1029	1173	4.00	0.77
ICCV 10	50	103	7527	4202	55.8	1275	1622	1.28	15.0	1041	1162	4.14	0.87

Mean	51.4	103.5	6925.6	3037.2	43.8	1420.1	1573.8	1.11	16.5	1059.0	1156.5	3.79	0.69
S.Ed (±)	0.54	1.92	381.3	89.87	1.89	129.6	119.3	0.06	0.78	9.24	49.6	0.25	0.03

↑Dv = Vegetative duration; Dr = Reproductive duration; C = Crop growth rate; p = Partitioning coefficient.

## Appendix A Drought treatment and drought treatment × genotype variances for the shoot traits measured at different days after sowing (DAS) in 12 diverse genotypes of chickpea grown in a Vertisol during 2009–10 and 2010–11 post-rainy season

↑SBM = Shoot biomass; SLA = Specific leaf area; LAI = Leaf area index; CTD = canopy temperature depression; Dr = Reproductive duration (°Cd); C = Crop growth rate; p = Parititioning coefficient.
