# Supplementary material for: Shoot traits and their relevance in terminal drought tolerance of chickpea (Cicer arietinum L.)
Source: Field Crops Res. 2016 Oct;197:10–27. doi: 10.1016/j.fcr.2016.07.016 (PMC5035057; doi:10.1016/j.fcr.2016.07.016)
Supplement: Supplementary file 1 [file mmc1.docx]

**Supplementary Figure S1:** Changes in available soil moisture up to a soil depth of 1.2 m across the crop growing seasons of 2009-10 and 2010-11. Vertical bars denote standard error of differences (±).

**Supplementary Figure S2:** Weather during the crop growing seasons (November to March) of 2009-10 and 2010-11.
